# Biosorption of Cadmium by Filamentous Fungi Isolated from Coastal Water and Sediments

**DOI:** 10.1155/2018/7170510

**Published:** 2018-10-22

**Authors:** Lebeth C. Manguilimotan, Jayzon G. Bitacura

**Affiliations:** Department of Biological Sciences, Visayas State University, Visca, Baybay City, Leyte 6521-A, Philippines

## Abstract

The use of microorganisms in decontaminating the environment encumbered with heavy metal pollutants through biosorption is considered as a good option for bioremediation. This study was conducted to isolate Cadmium (Cd) tolerant fungi from coastal waters and sediments, compare their biosorption capabilities, and identify the isolates with the highest Cd uptake. Water and sediment samples were collected near the effluent sites of industrial belt in Ibo, Lapu-lapu City, Cebu, Philippines. Potato dextrose agar (PDA) plates containing Cd (25, 50, 75, and 100 ppm) were used to isolate Cd tolerant fungi from the samples. The distinct colonies that grew on the highest Cd concentration (100 ppm) were then isolated into pure cultures. The pure cultures of Cd tolerant fungi served as a source of inocula for* in vitro* biosorption assay using Cd dissolved in potato dextrose broth (PDB) as the substrate. Cd tolerant fungal isolates with the highest Cd uptake were finally identified up to the lowest possible taxon based on their colonial and microscopic characteristics. Most filamentous fungal colonies have grown most at the lower Cd concentrations and least at the higher concentrations. From the characteristics of the fungal growth on the plate with the highest Cd concentration, eight distinct colonies from both sediment and water samples were isolated into pure cultures. Among the eight fungal isolates, only three had significant Cd biosorption efficiency, these were fungal isolate 3 (13.87 %), fungal isolate 6 (11.46 %), and fungal isolate 4 (10.71 %). Two of them (fungal isolates 3 and 4) belong to genus* Aspergillus *while the other (fungal isolate 6) is a species of* Penicillium*. The results of this study showed that Cd tolerant fungi with biosorption capacity could be isolated from coastal water and sediments in the vicinity of areas suspected of heavy metal contamination.

## 1. Introduction

Heavy metals are one of the constituting pollutants in water which are on the forefront of academic and regulatory concerns today. Metal effluents from the metal processing industries that are discharged into water bodies are not biodegraded but undergo chemical or microbial transformations, creating a large impact on the environment and public health [1, 2]. Recently, they have been found to be negatively affecting the gamete viability, fertilization, and embryonic development of* Tripneustes gratilla*, a marine invertebrate model organism [3]. Awareness of the importance of treatments and removal of heavy metals from such effluents to permissible limits before discharging into natural streams, rivers, and seas is rapidly growing worldwide.

Towards this direction, several conventional wastewater technologies were developed and are in use successfully at a large scale to reduce hazardous compounds concentration in wastewaters [4]. However, application of such traditional treatments is not economical. It requires continuous inputs of chemicals and it causes further environmental damages making it impractical. Hence, easy, effective, economic, and eco-friendly techniques are required for fine-tuning of wastewater management.

One of the identified reducing agents for heavy metals is the use of microorganisms like fungi [5–8]. Fungi can tolerate and detoxify metals in many ways. It could be through valence transformation, active uptake, precipitation inside or outside their cells, and biosorption [9–17]. Biosorption is a process of metal uptake by living or dead biomass through the binding of metal ion on the cell wall and extracellular materials [7].

The high surface-volume ratio of microorganisms and their ability to detoxify metals are among the reasons that they are considered as a potential alternative to synthetic resins for remediation of dilute solutions of metals and solid wastes [18]. The use of fungi, for instance, gained importance because it is eco-friendly, economical, and effective [19]. The cell wall of fungi consists of polysaccharides and proteins that offer multiple active sites for binding of metals [20]. The polysaccharides found in the cell walls of fungi are chitin and chitosan, which have been shown to sequester metal ions. The first step in biosorption is passive biosorption that occurs independently of cellular metabolism and proceeds rapidly by metal binding mechanisms like ion exchange, physical adsorption, coordination, complexation, or inorganic microprecipitation. Passive biosorption is a reversible adsorption-desorption process. Elution could be done by other ions, chelating agents, or acids. Conversely, active biosorption occurs when metal ions penetrate the cell membrane and enter into the cells [20, 21].

Considering the mechanisms of metal resistance by fungi, it is expected that screening of metal tolerant fungi may provide strains with improved metal accumulation. Only limited studies have been conducted in the Philippines to systematically screen filamentous fungi from metal-polluted sites for their metal tolerance. Therefore, the isolation, characterization, and identification of Cadmium (Cd) tolerant indigenous fungi with biosorption potential from the coastal waters and sediments near the industrial plants in Barangay Ibo, Lapu-Lapu City, Cebu, Philippines, was made. Cd is one of the known environmental pollutants that are frequently encountered together in sewage and industrial wastewaters [22]. Hence the metal was chosen for this study.

## 2. Materials and Methods

### 2.1. Collection of Samples

Composite sediment and coastal water samples were collected 10 meters away from the effluent sites of the industrial plants in Barangay Ibo, Lapu-Lapu City, Cebu. A hand corer was used to obtain the sediment samples. Samples were obtained by pushing the corer up to 10 cm depth in the sediments. A water sampler was also used to collect coastal water samples. The water sampler was towed horizontally 5 meters away from the shore. Five replicates of water and sediment samples were collected at every sampling point. Three sampling points were considered in the site. These points are at least 100 meters apart.

The collected sample replicates from each sampling point were mixed thoroughly for uniform distribution of the fungal cells. From these composite samples, approximately 500 grams of sediments was placed in a sterile 500 mL glass beaker and was covered with aluminum foil [23] while approximately 1.25 L of water sample was placed in sterile ketchup bottles. Sediments and water samples were placed in an ice bucket and were transported immediately to the laboratory for microbiological analyses that were carried out within 48 hours after sample collection.

### 2.2. Isolation of Cadmium Tolerant Fungi

All the glassware used in the experiment was acid washed to avoid unwanted metal contamination. In a 250 mL beaker 10 g of composite sediment sample was suspended in 90 ml of sterilized distilled water and was shaken for 30 min. One mL (1 mL) of the diluted soil suspension and the water sample was spread plated on the previously prepared sterile potato dextrose agar (PDA) plates containing increasing cadmium (Cd) concentrations of 25 ppm, 50 ppm, 75 ppm, and 100 ppm. These concentrations fall in the range of the minimum inhibitory concentrations of some filamentous fungi on certain heavy metals [8]. On the other hand, plates with no Cd served as the negative control. The inoculated plates were incubated at 28°C for seven days. The morphologically distinct colonies that grew on the highest Cd concentration were considered as Cd tolerant fungi. According to the review of Igwe and Abia [24], biosorbents are inefficient when heavy metal concentrations reach 100 mg/L or 100 ppm. Thus, fungal colonies growing on these Cd concentrations could be more tolerant to Cd than those that grew at lower Cd levels. The colonies were purified on plates and slants of potato dextrose agar (PDA). These pure cultures served as a source of inoculums for the biosorption assay and for further characterization and identification.

### 2.3. Biosorption Assay

Prior to the assay, fungal isolates were grown for a week in a potato dextrose broth (PDB). Each of the isolates was assayed by adding 20 mL of the previously grown fungal biomass in sterile 250 mL Erlenmeyer flasks containing 100 mL of the previously prepared sterile PDB with 10 mL CdSO_4_. Three replicates for every treatment were prepared. Three flasks containing CdSO_4_ were not inoculated with fungal biomass serving as the negative controls. The initial concentration of Cd in the experimental and control flasks was first determined then the flasks were incubated at room temperature with constant shaking for five days [8]. After the incubation, the media from the inoculated flasks were centrifuged to separate the fungal mycelia from the broth. The supernatants produced after centrifugations were then analyzed for dissolved Cd.

The initial and final Cd concentrations were determined through Atomic Absorption Spectrophotometry (AAS) at the Central Analytical Services Laboratory of the PhilRootcrops Research and Training Center, Visayas State University. From the data gathered, the biosorption efficiencies of the fungal isolates were then evaluated using the equation below [25].

(1) $$\begin{array}{c} E=\left[\;\frac{\left(Ci-Cf\right)}{Ci}\;\right]x\;100 \end{array}$$

where

  E is biosorption efficiency (%)
  Ci is initial concentration of the metal in the solution
  Cf is final concentration of the metal in the solution

### 2.4. Characterization and Identification of Cd Tolerant Fungal Isolates

The fungal isolates with the highest percentage of metal uptake were characterized and identified based on their colony shape, color, and spore formation as well as the texture of fungal growth. Additionally, the microscopic characteristics of the isolates, like their conidia, conidiophores, and hyphae, were observed under an electric compound microscope (True Vision Microscope USA) at high power objective (400X) and oil immersion objective (1000X). Canon PowerShot A2200 digital camera was used to document the cell and colony morphology of the Cd tolerant fungal isolates.

### 2.5. Experimental Design and Statistical Analysis

The biosorption efficiency of the Cd tolerant fungal isolates was evaluated in Completely Randomized Design (CRD). Analysis of Variance (ANOVA) was used to determine the significant difference on the biosorption efficiencies of the Cd tolerant fungal isolates followed by post hoc multiple comparisons of means using Tukey's Honestly Significant Difference (HSD) Test to determine the isolates that show significant biosorption efficiencies.

## 3. Results and Discussion

**Figure 1:**
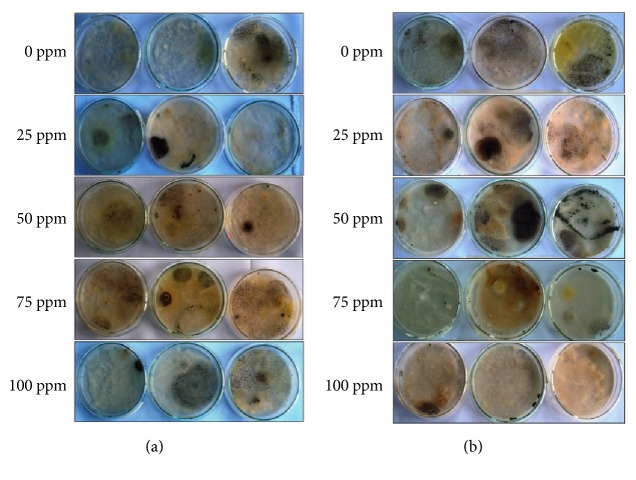
Seven-day-old fungal colonies from (a) water samples and (b) sediment samples isolated on PDA plates with 0 ppm, 25 ppm, 50 ppm, 75 ppm, and 100 ppm Cd concentrations.

Fungal colonies have grown on all plates containing different Cd concentrations. Most colonies have grown on lower Cd concentrations and least at higher concentrations (Figure 1). Five distinct colonies of fungi were isolated from the sediment samples and four distinct colonies of fungi from the water samples. However, after the pure culture, one isolate is found to have been isolated from both water and sediment samples, thus, giving a total of eight distinct fungal isolates.

**Figure 2:**
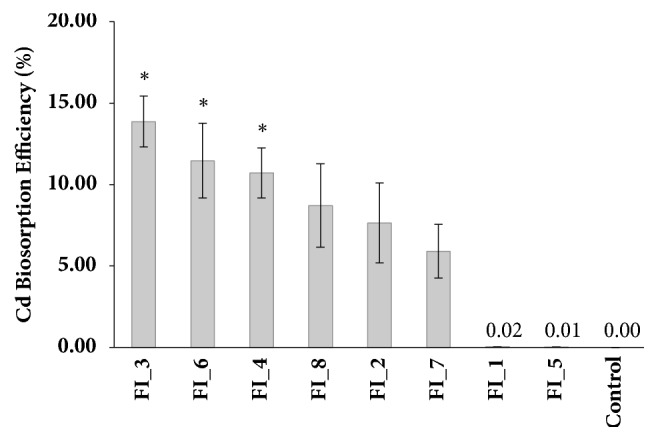
Cd biosorption efficiency (mean % ±SE; n=3) of the fungal isolates after five days of biosorption assay; ^*∗*^(p ≤ 0.05) Tukey's HSD; FI=fungal isolate.

Upon subjecting the eight fungal isolates to biosorption assay, it was found out that there was a significant difference in the biosorption efficiencies of the isolates (p ≤ 0.05). Post hoc comparison of mean Cd biosorption efficiency revealed that fungal isolate 3 (13.87 %), fungal isolate 6 (11.46 %), and fungal isolate 4 (10.71 %) have significant biosorption capacities among all the fungal isolates. Following closely were fungal isolate 8 (8.71 %), fungal isolate 2 (7.64 %), and fungal isolate 7 (5.90 %), while fungal isolates 1 and 5 showed the least biosorption efficiencies at 0.02 % and 0.01 %, respectively (Figure 2).

**Figure 3:**
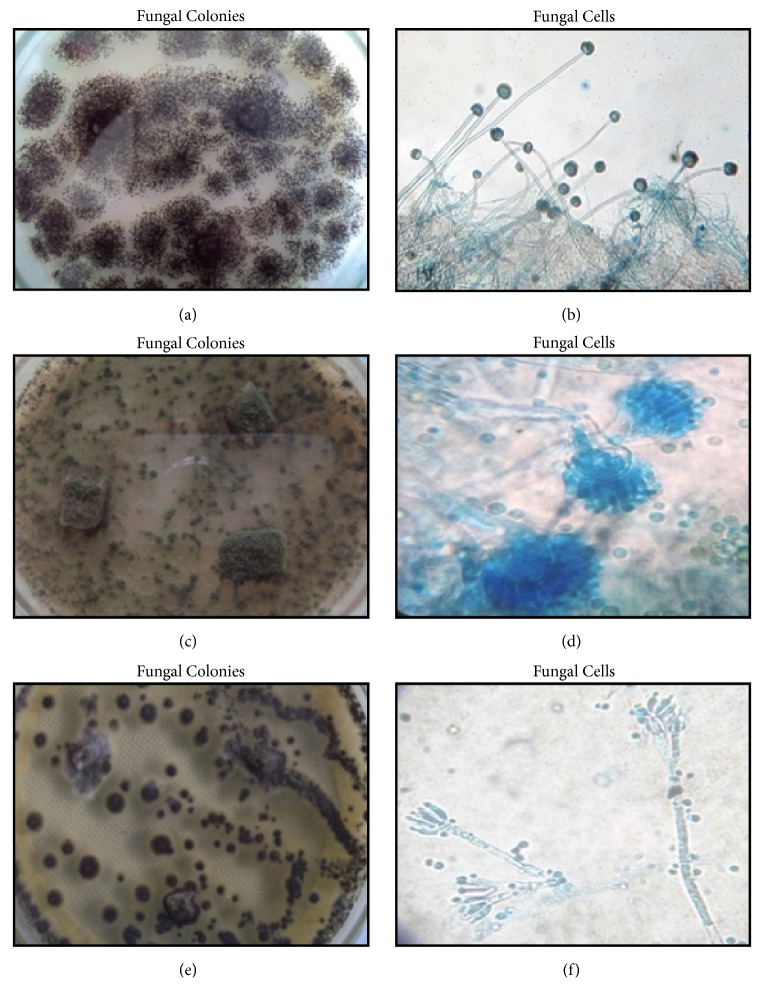
Colonial (left) and microscopic (right) characteristics (400X) of the three Cd tolerant fungal isolates with the highest percentage of Cd biosorption efficiency. Fungal isolates 3 (a and b) and 4 (c and d) were identified as* Aspergillus* spp. (a-d) while fungal isolate 6 (e and f) was identified as* Penicillium* sp.

Figure 3 shows the colonial and microscopic characteristics of the fungal isolates with the highest Cd uptake. Fungal isolate 3 consists of a compact dense layer of dark-brown to black colonies which are patching (Figure 3(a)). Conidial heads were short columnar and biseriate with the phialides borne on brown, septate metulae. Their conidiophores were turning dark towards the vesicle (Figure 3(b)). These are the characteristics of fungi belonging to genus* Aspergillus*. Colonies of fungal isolate 4 have a powdery texture. They were brownish green in color and became brownish-black as they aged. Their growth produced pale green exudates in the agar (Figure 3(c)). Microscopically, their conidial heads were radiating and columnar which form a brush-like structure (Figure 3(d)). This means that fungal isolate 4 belongs to the genus* Aspergillus* too. Finally, fungal isolate 6 colonies were fast growing; they were in shades of dark green and consist of dense felt conidiophores (Figure 3(e)). Microscopically, they have conidiophores that are hyaline and have a three-stage branch. They also have simple single-celled conidia (ameroconidia) in chains that were produced and attached in the basipetal succession of phialides, specialized conidiogenous cells giving the brush-like appearance of the species (Figure 3(f)). These characteristics point to the genus* Penicillium*.

The occurrence of various filamentous fungi in sediments with heavy metals has also been reported in other works from different parts of the world [26, 27]. It may be because sediments contain nutrients and provide substrate needed for the growth of the vegetative hyphae of these filamentous fungal species. The sediments may also provide a place for microorganisms to hide against environmental changes, making them more stable. On the other hand, less fungal species were isolated in the water samples may be attributed to instability of water.

The variations in the Cd uptake among the different fungal biomass of different species may be related to their chemical characteristics [20]. Biosorption of this metal is based on ions associating with the cell surface wherein ion exchange and complexation reaction with functional groups like carboxyl, amides, hydroxyl, phosphate, and sulphydryl groups occur [8]. Kapoor and Viraraghavan [28] reported that carboxylate and amine groups are important in metal ion biosorption on by some filamentous fungal species.

Biosorption of heavy metals by fungi isolated from wastewater-contaminated sites have been reported by other researchers too. Faryal et al. [29] isolated filamentous fungi from the soil of the local textile industry. They were able to isolate* Aspergillus *sp.*, Rhizopus *sp.*, Rhodotorula *sp.*, Drechslera *sp., and* Curvularia *sp. Parameswari et al. [8] isolated* Aspergillus niger*,* Phanerochaete chrysosporium*, and* Trichoderma viride* from municipal sewage contaminated with heavy metals. Among their isolates,* P. chrysosporium* accumulated 64.25 % of Cr and 57 % of Ni. Javaid and Bajwa [30] reported that* Pleurotus ostreatus* has 55% of biosorption efficiency for Cr (III) ions while Javaid et al. [25] revealed that* Schizophyllum commune* has biosorption efficiency of 72.01, 53.16, 7.08, and 19.87% for Cu (II), Ni (II), Zn (II), and Cr (VI) ions, respectively. These studies thus imply that biosorption efficiency of a certain fungal species may depend on the type of heavy metals they are subjected to. For instance, Khattab [31] reported that* Penicillium viridicatum *had high extraction activity for Pb but it had low dissolution activity for Zn and Cu from effluents.

Finally, biosorption of toxic metals depends on the extent of metabolic dependence [32]. The physiological state of the organism, the age of the cells, the availability of micronutrients during their growth, and the environmental conditions during the biosorption process (such as pH, temperature, and the presence of certain co-ions) are important parameters that affect the performance of a living biosorbent [33].

## 4. Conclusion

This study has shown that Cd tolerant fungi with biosorption capacity can be isolated from areas suspected of Cd contamination. Although further studies need to be done in order for these isolates to be used in bioremediation initiatives, this study opens a new venue of low cost and eco-friendly management of heavy metal pollutants.

## Data Availability

The data used to support the findings of this study are available from the corresponding author upon request.
